# Progression detection capabilities of circumpapillary and macular vessel density in advanced glaucomatous eyes

**DOI:** 10.1038/s41598-022-16083-9

**Published:** 2022-07-15

**Authors:** Anna Lee, Kyung Rim Sung, Joong Won Shin

**Affiliations:** Department of Ophthalmology, College of Medicine, University of Ulsan, Asan Medical Center, 388-1 Pungnap-2-dong, Songpa-gu, Seoul, 138-736 Korea

**Keywords:** Neuroscience, Diseases

## Abstract

This study investigated the progression detection capabilities of circumpapillary and macular vessel density (cpVD and mVD) in advanced primary open angle glaucoma (POAG) eyes using the rates of change in VD (trend-based analysis) and variability limits derived from healthy eyes. (event-based analysis) This study included 75 POAG eyes [visual field (VF) mean deviation < − 10 decibels, mean follow-up; 2.3 years] and 33 healthy eyes. Of 75 POAG eyes, 17 (22.7 %) and 58 eyes (77.3 %) were classified into the VF progression and stable groups, respectively. The VF progression group showed significantly faster VD loss than the stable group. (cpVD; − 1.76 vs. − 0.84 %/year, mVD; − 1.10 vs. − 0.47 %/year, P < 0.05) However, the rates of change in circumpapillary retinal nerve fiber layer and macular ganglion cell complex thickness were similar between the groups. (cpRNFLT; − 0.67 vs. − 0.53 $\mu$m/year, GCCT; − 0.48 vs. − 0.12 $\mu$m/year, P > 0.05) Event-based analysis showed stronger agreement between VD and VF progression (cpVD; kappa value (k) = 0.630, mVD; k = 0.667, P < 0.05) than that between structure and VF progression. (cpRNFLT; k = 0.111, GCCT; k = 0.194, P > 0.05). In conclusion, VD loss showed better progression detection capabilities than structural loss in advanced POAG eyes. Detection of cpVD and mVD loss may be useful for detecting progression in the advanced stages of POAG to complement other reference standard strategies.

## Introduction

Glaucoma is defined as progressive optic neuropathy which accompanies characteristic visual field (VF) deterioration^1^. Detecting glaucomatous progression is essential to prevent irreversible vision loss. Generally, progressive retinal nerve fiber layer (RNFL) atrophy or optical coherence tomography (OCT) measured RNFL thinning earlier than the detectable reduction of sensitivity in standard automated perimetry (SAP)^2–4^. Therefore, OCT is currently the standard method for early detection and has been widely used for glaucoma monitoring based on structural abnormalities such as RNFL and macular ganglion cell-inner plexiform layer (GC-IPL) thinning. Unfortunately, however, detecting glaucomatous progression using OCT in advanced glaucoma is challenging because RNFL is not sensitive enough to detect glaucomatous progression as it already reaches the measurement floor^5,6^. Functional assessment usually fails to accurately detect glaucomatous progression due to greater VF fluctuation in the advanced stages^7–9^.

The recent introduction of OCT angiography (OCTA) has allowed clinicians to noninvasively image the microvasculature of the optic nerve head (ONH), retina, and choroid and evaluate the perfusion status of these structures^10–12^. Vessel density (VD) assessed by OCTA may be a useful method for diagnosing glaucoma and monitoring glaucomatous progression^13–15^. VD assessment showed good diagnostic performance, reproducibility and repeatability, which enhances reliability in progression monitoring^16^. In addition, circumpapillary and macular VD (cpVD and mVD) are less likely to reach the measurement floor than thickness parameters in advanced glaucoma, suggesting that VD assessment may be more useful than RNFL or GC-IPL measurements for detecting glaucomatous progression in advanced stages^17–20^.

Both event- and trend-based analyses are widely used to detect glaucomatous progression. Event analysis can detect glaucomatous progression with a relatively smaller number of tests compared to trend-based analyses. Further to this, in clinical practice, whether structural and functional loss exceeds the stable glaucoma population-derived variability limits is important in judging glaucoma progression. The trend-based approach has the advantage of providing the progression rate. In this study, we investigated the progression detection capabilities of circumpapillary and macular VD in advanced primary open angle glaucoma (POAG) eyes with the rate of change in VD measurements and via an event-based analysis using the variability limit derived from real-world data.

## Methods

### Study subjects

We retrospectively recruited participants by reviewing the medical records of patients who visited the glaucoma clinic at Asan Medical Center from January of 2017 to April of 2021. This study was approved by the Institutional Review Board of Asan Medical Center and is compliant with the Declaration of Helsinki. Informed consent from the study subjects was waived by the IRB of Asan Medical Center due to the retrospective study design.

Both healthy controls and POAG patients were recruited for the study. All participants underwent comprehensive ophthalmic examination, including a review of their medical history, measurement of best-corrected visual acuity (BCVA), intraocular pressure (IOP, Goldmann applanation tonometry), refractive error, axial length (IOL Master version 5; Carl Zeiss Meditec, Dublin, CA, USA), ultrasound pachymetry for central corneal thickness (CCT) (DGH-550; DGH Technology, Inc., Exton, PA, USA), and slit-lamp microscopy. POAG was diagnosed after an initial glaucoma workup including dilated fundus ophthalmoscopy, gonioscopy, optic disc stereophotography and red-free RNFL photography (Canon, Tokyo, Japan), VF testing with SAP (Humphrey field analyzer with Swedish interactive threshold algorithm standard 24–2 test; Carl Zeiss Meditec), spectral-domain optical coherence tomography (SD-OCT) (Spectralis HRA OCT; Heidelberg Engineering, Heidelberg, Germany), and OCTA (Angiovue; Optovue Inc., Fremont, CA, USA).

Inclusion criteria for both healthy and POAG patients were (1) age $\ge$ 18 years; (2) BCVA $\ge$ 20/30 and spherical equivalent between − 8.0 and + 3.0 diopters (D), and cylinder correction within $\pm$ 3 D; and (3) normal anterior chamber and open angle on slit lamp and gonioscopy. The study exclusion criteria were (1) any history of intraocular surgery (including any cataract/glaucoma), any history of trauma or laser treatment during follow-up; (2) severe media opacities, including cataracts of more than C2, N2, or P2 based on the Lens Opacities Classification System III^21^, which obscure the scan image; and (3) ocular diseases other than glaucoma, such as severe myopic maculopathy, diabetic retinopathy, retinal venous occlusive disease, optic neuritis, or systemic/neurologic disease that could influence the ONH and VF tests. Other exclusion criteria included unreliable VF results (fixation loss > 20%, false-positive error > 15%, and false-negative error > 15%) and poor-quality OCT/OCTA images (more details on these exclusion criteria are provided in the OCT/OCTA sections). If both eyes were eligible in any subject, one eye was randomly selected for the study.

Inclusion criteria for glaucoma patients were (1) the presence of a glaucomatous optic disc finding (i.e., the presence of focal neuroretinal rim thinning, notching, localized or diffuse atrophy of RNFL) and glaucomatous VF defects according to Anderson’s criteria as confirmed by at least two reliable VF examinations [three or more adjacent points on a pattern deviation probability map with P < 0.05 and one abnormal point with P < 0.01, a glaucoma hemifield test outside the normal limits, or a pattern standard deviation (PSD) of P < 0.05 on two consecutive reliable VF tests (false-positive errors < 15%, false-negative errors < 15%, and fixation loss < 20%)]^22^; (2) an average of the initial two VFs mean deviation (MD) measurements of less than − 10 decibels (dB) to include advanced-stage of POAG eyes; and (3) at least five serial VF tests and at least four serial SD-OCT and OCTA scans every 6–12 months during a minimum of two years. Patients with VF defects resulting from general, neurologic, or any other ophthalmic condition were excluded. The first VF result was excluded to obviate any learning effect.

Healthy controls were individuals who visited our glaucoma clinic for a regular health examination. They were matched to OAG eyes by age ($\le$ 10 years) and axial length ($\le$ 1 mm) and were required to have (1) an IOP < 21 mmHg with no history of elevated IOP; (2) normal appearance of the optic disc and an intact neuroretinal rim; (3) normal RNFL thickness (i.e., average and quadrant RNFL thickness within 99% confidence limits) based on SD-OCT; (4) normal VFs (i.e., a PSD within 95% confidence limits and a GHT result within the normal limit)^22^; and (5) OCT and OCTA scans at least three times every 6–12 months during the follow-up.

### Vessel density, ganglion cell complex thickness, and retinal nerve fiber layer thickness acquisition

All OCTA scans of macular and ONH were performed using the AngioVue OCTA system (software version 2018.1.0.43, Angiovue; Optovue Inc., Fremont, CA, USA) in the high-definition (HD) retinal, HD optic disc scan and ganglion cell complex (GCC) scan. This OCTA system utilized the split-spectrum amplitude-decorrelation angiography method to capture the dynamic motion of red blood cells and provide high-resolution three-dimensional visualization of vascular structures^23^.

Macular whole image vessel density measurements (mVD) were calculated from images of 6 × 6-mm^2^ scans centered on the fovea. An automated segmentation algorithm of the AngioVue software was applied to visualize the superficial retinal capillary plexuses [from the internal limiting membrane (ILM) to the posterior boundary of the inner plexiform layer (IPL)].

The capillary vessel was evaluated from images of 4.5 × 4.5-mm^2^ scans centered on the ONH within the radial peripapillary capillary slab from the ILM to the nerve fiber layer after automated removal of any large vessels. cpVD measurements were the percentage of measured area occupied by small vessels within an instrument-defined 1000-µm-wide elliptical annulus from the optic disc boundary.

Macular ganglion cell complex (GCCT) measurements consisting of the ganglion cell layer, IPL, and RNFL were obtained from a 6-mm-diameter area on the macular centered 1 mm temporal to the fovea.

Spectralis SD-OCT (Spectralis HRA OCT; Heidelberg Engineering, Heidelberg, Germany) was used to measure circumpapillary RNFL thickness (cpRNFLT). Spectralis uses a dual-beam, a confocal laser-scanning ophthalmoscopy, to acquire reference scans for eye-movement tracking and OCT images. cpRNFLT was measured from a high-resolution 3.46-mm-diameter circular scan centered on the optic disc.

All participants underwent OCT, OCTA and VF tests on the same day during follow-up. Three glaucoma specialists (A.L., J.W.S., and K.R.S.) reviewed all OCT and OCTA images to evaluate image quality. OCT/OCTA images were excluded if they had (1) poor image quality with quality score < 12 for OCT scans and a signal strength index < 30 for OCTA scans; (2) motion artifacts (i.e., significant residual motion line); (3) localized weak signal intensity caused by vitreous floaters; (4) poor clarity (i.e., media opacity); (5) fixation error; or (6) segmentation failure.

### Assessment of glaucoma progression

#### Visual field progression

Only reliable VFs with $\le$ 15% false positives, $\le$ 15% false negatives, and $\le$ 20% fixation loss were included in the study. Progression was defined if there was a significant deterioration from the baseline pattern deviation at more than three of the same test points evaluated on three consecutive examinations based on the Early Manifest Glaucoma Trial (EMGT) criteria (event-based analysis)^24^. Only “likely progression” was defined as VF progression. In the case which had too advanced VF MD to detect VF progression based on the EMGT criteria (VF MD < − 16 dB), VF progression was defined if the linear regression of the MD slope was negative with a P-value less than 0.05. (trend-based analysis) Each eye was classified in the VF progression or stable groups, respectively.

#### OCT/OCTA-derived parameter progression

VD, cpRNFLT, and GCCT progression were defined if two consecutive OCT/OCTA tests decreased more than the tolerance limit compared with the baseline test. For this event-based analysis, the tolerance limit was obtained using the longitudinal variability measurements in healthy eyes, which were the same as the within-subject standard deviation (Sw) from a single healthy eye if tested multiple times. The tolerance limit was defined as 1.96 × $\surd 2$ × Sw. Additionally, we determined the intraclass correlation coefficient (ICC) and coefficient of variation (COV) to assess reproducibility. We calculated ICC by the two-way random effects model using the absolute agreement definition. The COV, expressed as a percentage (%), was calculated as the square root of the variance divided by the mean of repeated measurements. ICC values ranged from 0–1, where 0 indicates no agreement and 1 perfect agreement between repeated measurements^25^.

The rate of changes in VD, cpRNFLT, GCCT, and VF MD over time were estimated by the trend-based approach using age-adjusted linear mixed-effects model analysis with random intercepts to account for within-participant variability.

### Statistical analysis

The distribution normality was assessed with the Kolmogorov–Smirnov test. Results are demonstrated as a mean and standard deviation or frequency and percentage. The demographic and clinical characteristics of the study subjects were compared between the VF progression and stable groups and the healthy control group. Comparisons among groups were performed using a one-way analysis of variance with Tukey’s post hoc test for quantitative variables. Fisher’s exact test was used for categorical variables.

Agreement between VF progression and OCT/OCTA-derived parameter progression was assessed by Kappa (k) statistics. The strength of agreement was categorized per Landis and Koch^26^: 0 = poor, 0–0.20 = slight, 0.21–0.40 = fair, 0.41–0.60 = moderate, 0.61–0.80 = substantial, and 0.81–1.00 = almost perfect. All statistical analyses were performed using SPSS ver. 21.0 program (SPSS Inc., Chicago, IL, USA). P-values less than 0.05 were considered statistically significant.

## Results

### General participant characteristics

After an initial review, we included 132 POAG eyes of 132 subjects who met the initial inclusion criteria (92 POAG eyes of 92 participants and 40 healthy eyes of 40 participants). Of these, we excluded 11 eyes with unreliable VF tests, 10 eyes with poor OCT/OCTA scan quality, one eye with proliferative diabetic retinopathy, and two eyes with epiretinal membranes. Therefore, 108 eyes consisting of 75 advanced POAG eyes and 33 healthy eyes were included in the final analysis. Mean follow-up duration and the number of visits were 2.3 $\pm$ 1.0 years and 4.5 $\pm$ 1.0 visits, respectively, for glaucoma eyes and 1.8 $\pm$ 0.9 years and 3.3 $\pm$ 0.6 visits, respectively, for healthy eyes. The baseline VF MD of the 75 POAG eyes was − 16.39 $\pm$ 5.02 dB.

Table 1 summarizes the demographic and baseline clinical characteristics of the participants with POAG and healthy controls. Among the 75 POAG eyes, VF progression was detected in 17 eyes (22.7%) by either event (8 eyes, 10.7%) or trend analysis (9 eyes, 12.0%), and 58 eyes (77.3%) showed stable VF. All OCT/OCTA parameters were significantly lower in both VF progression and stable groups than in the healthy group (P < 0.05). There was no statistical difference between the VF progression and stable groups in all parameters, except for the number of glaucoma medications at the first visit (2.2 $\pm$ 0.7 vs. 1.2 $\pm$ 1.0, P < 0.001) and final VF MD (− 19.22 $\pm$ 5.98 vs. − 16.51 $\pm$ 4.42, P = 0.048).

**Table 1:** Demographics and ocular characteristics of the study population.

Parameters	POAG (n = 75)		C. Healthy (n = 33)	P-value (post hoc A vs B, A vs C, B vs C)
	A. Progression (n = 17)	B. Stable (n = 58)		
Age (years)	54.1 $\pm$ 13.6	54.4 $\pm$ 14.1	50.3 $\pm$ 16.8	0.427
Gender, male/female	13:5	30:28	18:15	0.447^a^
Hypertension, n (%)	2 (11.8)	9 (15.5)	5 (15.1)	0.929^a^
Diabetes mellitus, n (%)	1 (5.9)	6 (10.3)	2 (6.1)	0.723^a^
Refractive error (diopter)	− 3.67 $\pm$ 3.53	− 2.78 $\pm$ 3.41	− 2.92 $\pm$ 4.11	0.680
Axial length (mm)	25.10 $\pm$ 1.52	25.68 $\pm$ 2.24	25.04 $\pm$ 1.85	0.317
CCT ($\bf \it \bf {\varvec{\mu}}{{m}}$)	537.91 $\pm$ 39.13	528.73 $\pm$ 37.85	542.27 $\pm$ 41.06	0.292
IOP at the first scanning visit (mmHg)	15.11 $\pm$ 3.74	15.52 $\pm$ 3.56	16.54 $\pm$ 4.99	0.407
Number of medications at the first scanning visit	2.2 $\pm$ 0.7	1.2 $\pm$ 1.0	0.0 $\pm$ 0.0	** < 0.001** (**< 0.001, 0.002, < 0.001**)^b^
**VF measurement (dB)**				
Baseline VF MD	− 16.62 $\pm$ 6.31	− 16.32 $\pm$ 4.64	− 0.69 $\pm$ 1.23	** < 0.001** (0.966, < **0.001, < 0.001**)^b^
Final VF MD	− 19.22 $\pm$ 5.98	− 16.51 $\pm$ 4.42	− 0.66 $\pm$ 1.34	** < 0.001** (**0.048, < 0.001, < 0.001**)^b^
**cpRNFL measurement (**$\bf \bf \it {\varvec{\mu}}\mathbf{{m}}$**)**				
Baseline average thickness	54.9 $\pm$ 13.7	59.4 $\pm$ 12.9	91.4 $\pm$ 13.6	** < 0.001** (0.462**, < 0.001, < 0.001**)^b^
Final average thickness	54.2 $\pm$ 14.2	59.1 $\pm$ 13.2	89.4 $\pm$ 12.0	** < 0.001** (0.377**, < 0.001, < 0.001**)^b^
**Macular GCC measurement (**$\bf \bf \it {\varvec{\mu}}\mathbf{{m}}$**)**				
Baseline average thickness	67.5 $\pm$ 9.9	66.7 $\pm$ 8.8	91.1 $\pm$ 11.0	** < 0.001** (0.961**, < 0.001, < 0.001**)^b^
Final average thickness	67.6 $\pm$ 9.5	66.1 $\pm$ 9.6	91.0 $\pm$ 10.9	** < 0.001** (0.906**, < 0.001, < 0.001**)^b^
**OCTA vessel density measurement (%)**				
Baseline average cpVD	37.4 $\pm$ 9.3	37.1 $\pm$ 6.0	48.2 $\pm$ 5.3	** < 0.001** (0.977**, < 0.001, < 0.001**)^b^
Final average cpVD	32.6 $\pm$ 6.4	35.3 $\pm$ 6.6	47.6 $\pm$ 6.0	** < 0.001** (0.290**, < 0.001, < 0.001**)^b^
Baseline average mVD	37.7 $\pm$ 3.8	37.1 $\pm$ 4.1	45.7 $\pm$ 5.2	** < 0.001** (0.863, < **0.001, < 0.001**)^b^
Final average mVD	35.6 $\pm$ 3.9	36.1 $\pm$ 4.5	45.0 $\pm$ 4.9	** < 0.001** (0.908**, < 0.001, < 0.001**)^b^

*CCT* central corneal thickness, *cpRNFL* circumpapillary retinal nerve fiber layer, *cpVD* circumpapillary vessel density, *dB* decibels, *GCC* ganglion cell complex, *IOP* intraocular pressure, *MD* mean deviation, *mVD* macular vessel density, *n* number, *OCTA* optical coherence tomography angiography, *VF* visual field.

Boldface values indicates statistical significance.

^a^Fisher exact test.

^b^one-way analysis of variance with the Tukey’s post hoc test.

### Trend-based analysis

Table 2 demonstrates the comparison of the rate of VF MD and OCT/OCTA-derived parameters determined by age-adjusted linear mixed-effects models between the VF progression and stable groups. Both groups showed a significant reduction in cpVD and mVD (P < 0.05), but there was no significant reduction in cpRNFLT and GCCT (P > 0.05). The reduction rate of MD (− 1.01 dB/year vs. − 0.19 dB/year, P < 0.001), cpVD (− 1.76%/year vs. − 0.84%/year, P = 0.019), and mVD (− 1.10%/year vs. − 0.47%/year, P = 0.018) were faster in the VF progression group than in the stable group.

**Table 2:** Comparisons of rates of change in VF MD and OCT/OCTA-derived parameters between eyes with and without visual field progression in 75 POAG eyes using age-adjusted linear mixed-effects models.

Parameters	Progression			Stable			P-value†
	Estimates	95% CI	P-value*	Estimates	95% CI	P-value*	
VF MD (dB/yr)	− 1.01	− 1.37 to − 0.65	** < 0.001**	− 0.19	− 0.42 to 0.04	0.107	** < 0.001**
cpRNFLT (${\varvec{\upmu}}\mathbf{m}/\mathbf{y}\mathbf{r}$)	− 0.67	− 1.44 to 0.10	0.086	− 0.53	− 0.89 to − 0.17	0.412	0.904
GCCT (${\varvec{\upmu}}\mathbf{m}/\mathbf{y}\mathbf{r})$	− 0.48	− 1.28 to 0.30	0.200	− 0.12	− 0.75 to 0.50	0.686	0.467
cpVD (%/yr)	− 1.76	− 2.89 to − 0.64	**0.005**	− 0.84	− 1.29 to − 0.40	** < 0.001**	**0.019**
mVD (%/yr)	− 1.10	− 1.86 to − 0.35	**0.006**	− 0.47	− 0.84 to − 0.09	**0.015**	**0.018**

*CI* confidence interval, *cpRNFLT* circumpapillary retinal nerve fiber layer thickness, *cpVD* circumpapillary vessel density, *GCCT* ganglion cell complex thickness, *mVD* macular vessel density, *OCT* optical coherence tomography, *OCTA* optical coherecnet tomography angiography, *POAG* primary open angle glaucoma, *VF MD* visual field mean deviation, *yr* year.

Boldface values indicates statistical significance.

* Whether the mean rate of change is significantly different from 0.

^†^Whether the mean rate of change is significantly different between groups.

### Event-based analysis

All OCT/OCTA-derived parameters in the 33 healthy eyes showed excellent long-term reproducibility with high ICC values ranging between 0.926–0.991 (Table 3). The tolerance limits of cpRNFLT, GCCT, cpVD, and mVD were $\pm$ 6.50 $\mathrm{\mu m}$, $\pm$ 4.12 $\mathrm{\mu m}$, $\pm$ 5.16%, and $\pm$ 4.96%, respectively. Based on those tolerance limits, progression was determined by each cpRNFLT, GCCT, cpVD, and mVD measurement, respectively.

**Table 3:** Longitudinal variability measurement determined by OCT/OCTA-derived parameters in 33 healthy eyes.

Parameters	ICC (95% CI)	COV (%)	Tolerance limit
cpRNFLT (${{\upmu}}{m})$	0.991 (0.972—0.998)	3.08	6.50
GCCT (${{\upmu}}{m})$	0.947 (0.897- 0.975)	1.63	4.12
cpVD (%)	0.938 (0.889—0.968)	2.85	5.16
mVD (%)	0.926 (0.867- 0.961)	3.91	4.96

*CI* confidence value, *COV* coefficient of variation, *cpRNFLT* circumpapillary retinal nerve fiber layer thickness, *cpVD* circumpapillary vessel density, *GCCT* ganglion cell complex thickness, *ICC* intraclass correlation coefficient, *mVD* macular vessel density, *OCT* optical coherence tomography, *OCTA* optical coherecnet tomography angiography.

The tolerance limit was defined as 1.96 $\times \surd 2$ $\times$ within-subject standard deviation (Sw).

COV is calculated as 100 X Sw/overall mean.

**Figure 1:**
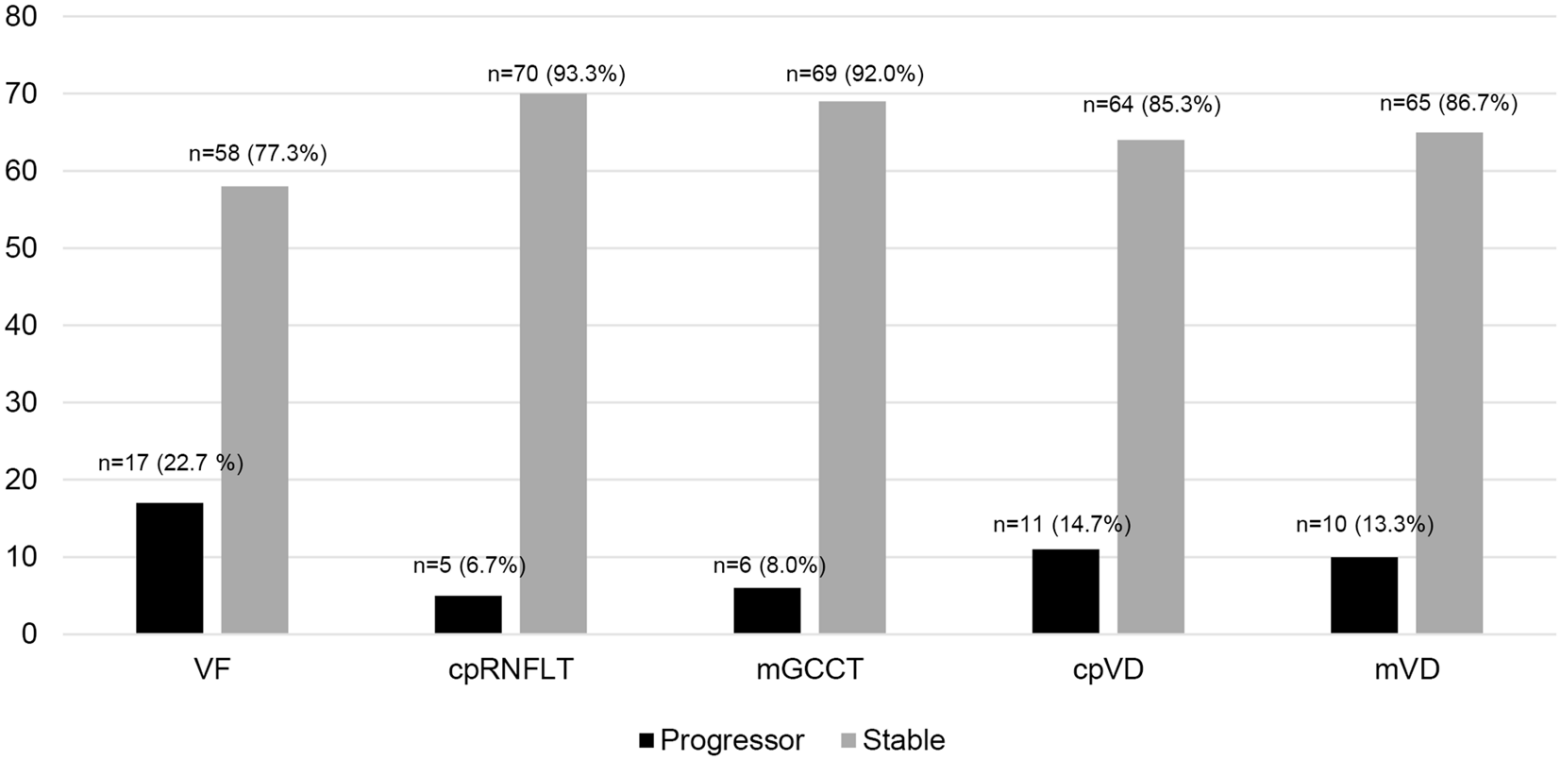
Histogram illustrating the number of eyes with progression (black bars) and stable eyes (gray bars). *cpRNFLT* circumpapillary retinal nerve fiber layer thickness, *cpVD* circumpapillary vessel density, *GCCT* ganglion cell complex thickness, *mVD* macular vessel density, *n* number, *VF* visual field,

The number of eyes with VF progression [n = 17 (22.7%)] was greater than that of cpRNFLT, GCCT, cpVD, and mVD progression [n = 5 (6.7%), n = 6 (8.0%), n = 11 (14.7%), and n = 10 (13.3%), respectively, Fig. 1]. Agreement between VF progression and OCT or OCTA-derived parameter progression was determined by kappa statistics (Table 4). The cpVD and mVD progressions showed substantial agreement with VF progression, with kappa values of 0.630 and 0.667 (P < 0.05), respectively. However, cpRNFLT and GCCT progression showed slight agreement with VF progression, with kappa values of 0.111 and 0.194 (P > 0.05), respectively.

**Table 4:** Agreement between VF progression and OCT/OCTA-derived parameters’ progression determined by the event-based analysis using kappa agreement statistics.

Parameters	k	P-value	Agreement
cpRNFLT	0.111	0.120	Slight
GCCT	0.194	0.055	Slight
cpVD	0.630	** < 0.001**	Substantial
mVD	0.667	** < 0.001**	Substantial

*cpRNFLT* circumpapillary retinal nerve fiber layer thickness, *cpVD* circumpapillary vessel density, *GCCT* ganglion cell complex thickness, *k* kappa value, *mVD* macular vessel density, *OCT* optical coherence tomography, *OCTA* optical coherecnet tomography angiography, *VF* visual field.

0 to 0.20 = slight; 0.21 to 0.40 = fair; 0.41 to 0.60 = moderate; 0.61 to 0.80 = substantial; 0.81 to 1.00 = almost perfect.

Among the 17 eyes which showed VF progression, eight showed VD reduction when VF progression was detected. In one patient (12.5 %), both cpVD and mVD reductions were detected on the same day visual field progression was detected. Five eyes (62.5 %) showed both mVD and VF progression at the same visit. and this was higher than those showing concurrent cpVD and VF progression. (two eyes, 25.0 %).

### Representative cases

**Figure 2:**
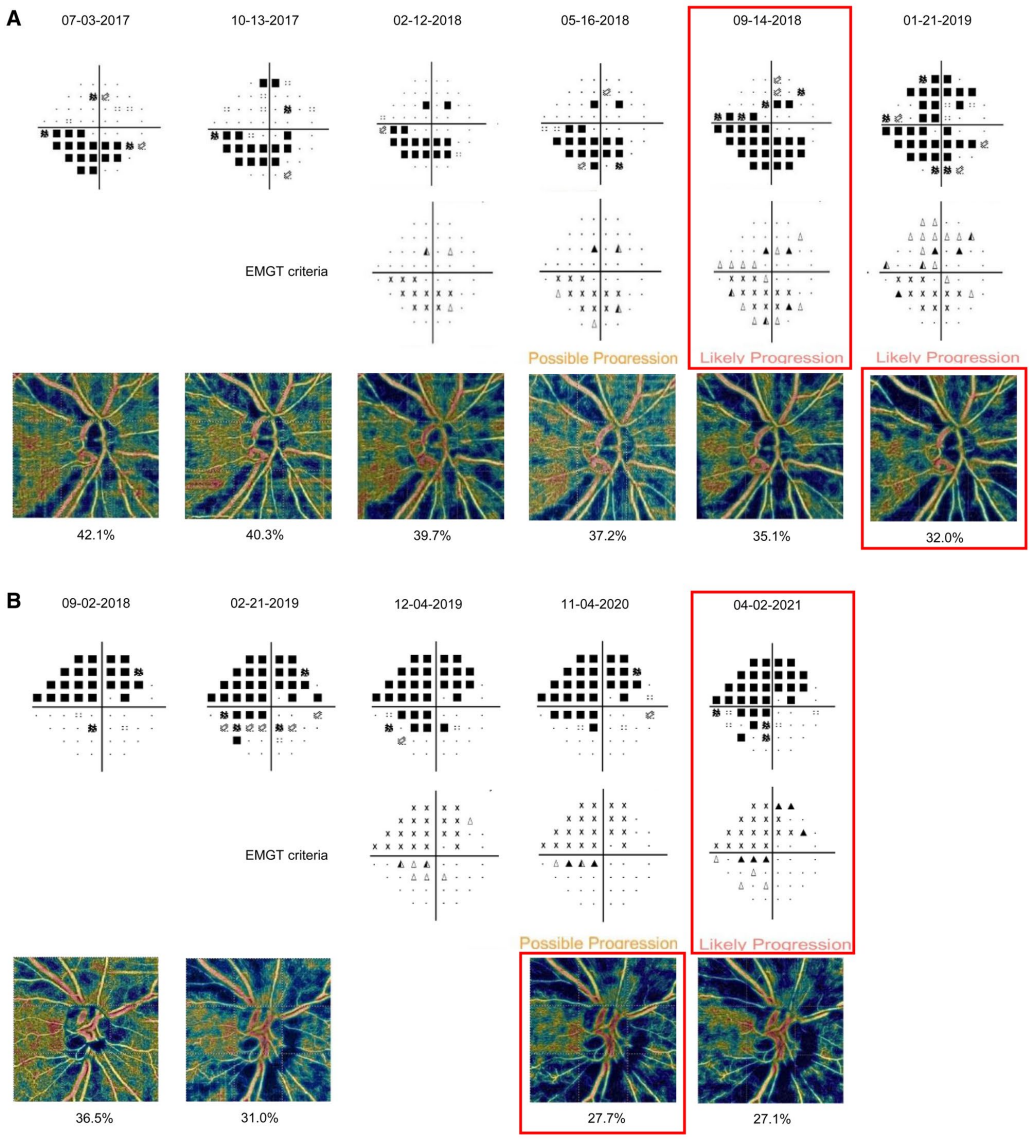
Two representative cases (**A**, **B**) with primary open-angle glaucoma showing progressive reduction of circumpapillary vessel density (cpVD) and visual field (VF) progression. (**A**) Representative case of a 76-year-old man with a VF mean deviation of − 10.95 dB at baseline demonstrated VF progression preceded by cpVD reduction. Significant progressive VF progression (“likely progression”) was detected on September 14, 2018. Approximately four months later, two consecutive optical coherence tomography angiography (OCTA) tests (on September 14, 2018, and January 21, 2019) showed cpVD reduction $\ge$ the tolerance limit (5.16 %) compared to baseline cpVD. B. Representative case of a 72-year-old female with VF MD, − 13.43 dB at baseline whose cpVD significantly decreased before VF progression was detected. Two consecutive OCTA tests on February 21, 2019, and November 4, 2020, showed cpVD reduction $\ge$ the tolerance limit (5.16 %) compared to the baseline cpVD. VF progression was detected on April 2, 2021. *cpVD* circumpapillary vessel density*, dB* decibel*, EMGT* Early Manifest Glaucoma Trial, *MD* mean deviation, *OCTA* optical coherence tomography angiography, *VF* visual field.

**Figure 3:**
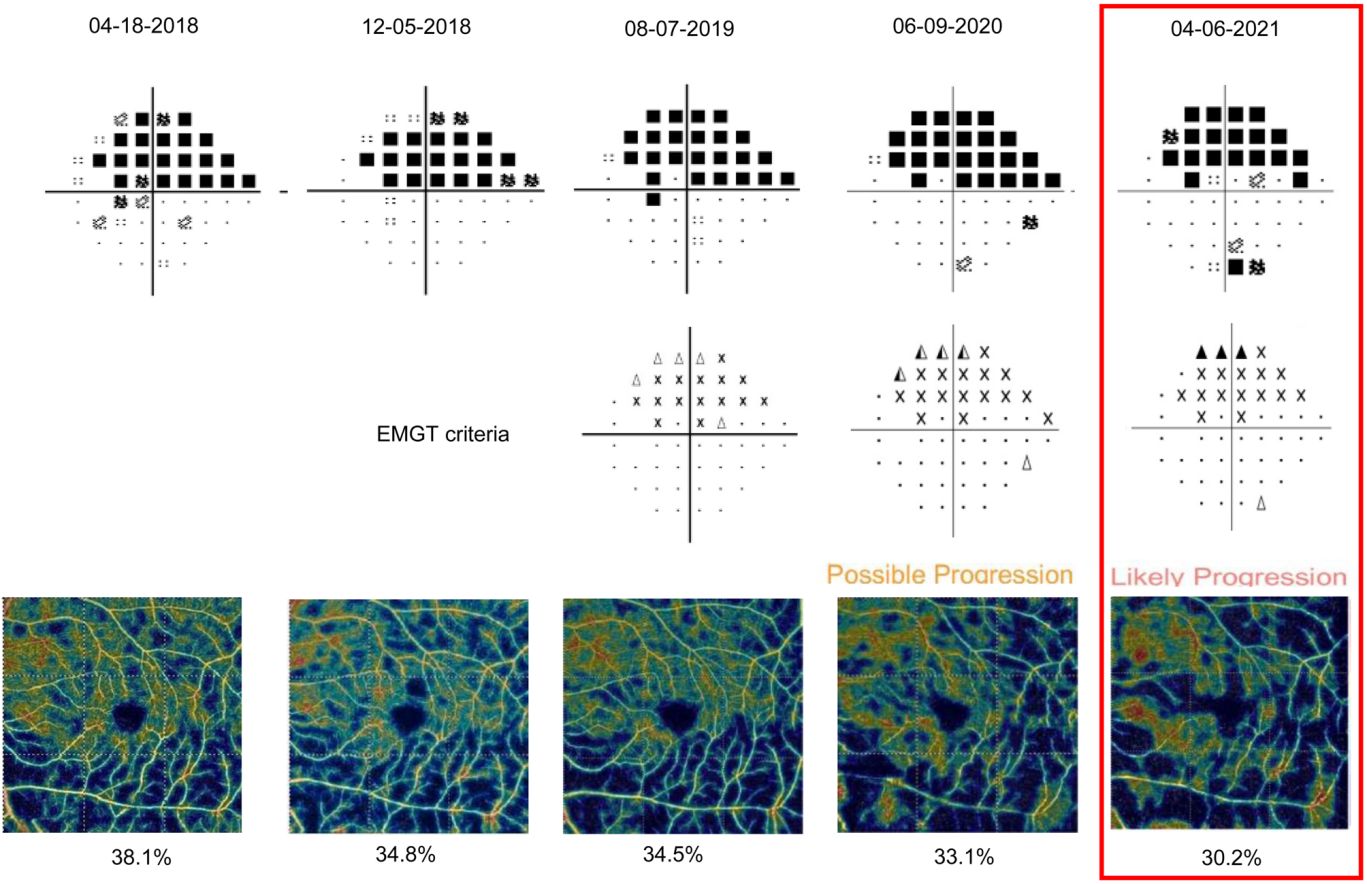
Representative case of a 69-year-old female [baseline visual field (VF) mean deviation (MD), − 11.46 decibels (dB)] showed macular vessel density (mVD) reduction [“two consecutive optical coherence tomography angiography (OCTA) showed mVD reduction $\ge$ the tolerance limit (4.96%) compared to baseline mVD”] compared to baseline mVD and significant VF progression (“likely progression”) on April 6, 2021. *dB* decibel*, EMGT* Early Manifest Glaucoma Trial, *MD* mean deviation, *mVD* macular vessel density, *OCTA* optical coherence tomography angiography, *VF* visual field.

Figures 2 and 3 show representative cases. In Fig. 2A, a 76-year-old man with baseline VF MD, -10.95 dB at baseline, demonstrated VF progression preceded by cpVD reduction. Significant VF progression (“likely progression”) was detected on September 14, 2018. Approximately four months later, two consecutive OCTA tests (on September 14, 2018, and January 21, 2019) showed cpVD reduction $\ge$ the tolerance limit (5.16 %) compared to baseline cpVD. Figure 2B demonstrates a 72-year-old female with baseline VF MD, − 13.43 dB at baseline who showed significantly decreased cpVD before VF progression was detected. Two consecutive OCTA tests on February 21, 2019, and November 4, 2020, showed cpVD reduction $\ge$ the tolerance limit (5.16 %) compared to baseline cpVD. VF progression (“likely progression”) was detected on April 2, 2021, approximately five months later. In Fig. 3, a 69-year-old female (baseline VF MD, − 11.46 dB) showed mVD reduction (“two consecutive OCTA showed mVD reduction $\ge$ the tolerance limit [4.96 %] compared to baseline mVD”) and significant VF progression (“likely progression”) on April 6, 2021.

## Discussion

Detecting progression in advanced glaucomatous eyes is a major challenge in glaucoma management. Previous studies have assessed the ability of VD to detect progression in advanced stage glaucoma by calculating the long-term rate of change of VD (trend-based analysis)^19,20^. However, in clinical practice, whether structural and functional loss exceeds the population-derived variability limits (event-based analysis) is important in determining glaucoma progression. Nonetheless, only limited information was available on longitudinal VD loss in respect to the event-based analysis in advanced POAG. Therefore, our objective was to evaluate the progression detection capability of VD parameters compared with OCT-derived parameters in advanced stages of glaucoma using both trend-and event-based analyses.

Our trend-based analysis showed that the perimetrically progressed group had faster rates of cpVD and mVD loss than the stable group in advanced glaucomatous eyes, whereas there was no significant difference in the cpRNFLT and GCCT thinning rates between the two groups (Table 2). In addition, the event-based analysis showed substantial agreement between VF progression and both cpVD and mVD progression. On the contrary, both cpRNFLT and GCCT progression showed weaker (“slight”) agreement with VF progression (Table 4). These findings are consistent with previous studies which revealed that thickness parameters are already close to the end of the dynamic range, but OCTA-measured VD does not approach a measurement floor until an advanced stage of glaucoma. The dynamic range of OCTA-measured VD is larger than that of the thickness parameters^6,17,27,28^. The measurement floor of cpRNFLT is around − 10 to − 15 dB of VF MD and that of GCCT is around − 8.3 to − 13.9 dB of VF MD^6,17,28–30^. After reaching the measurement floor, thickness parameters are no longer sensitive enough to detect glaucomatous progression.

Our previous study and others have reported that macular GCCT measurements are superior to cpRNFLT measurements in monitoring glaucomatous progression^5,31,32^. However, GCCT was not as sensitive as VD measurements in our results, which may be because our current POAG eyes were at a significantly more advanced (MD = − 16.39 dB) stage than our previous study^31^ (MD = − 12.27 dB). Moghimi et al.^17^ reported that the OptoVue-measured GCC floor was 70.7 $\mathrm{\mu m}$ thick and VF MD at the estimated GCC floor was − 13.9 dB. The baseline GCCT and VF MD of the current study exceeded these measurement floors. However, several study results differed from our study result. Bowd et al.^5^ measured the mean rates of change in GCIPL thickness in 87 eyes with advanced glaucoma (MD = − 17.0 dB) using Spectralis SD-OCT and found them to be − 0.21 $\mu$m/y (P < 0.001), which was significantly different from zero. Belghith et al.^32^ measured the mean rate of GC-IPL change using Spectralis SD-OCT and found that it reached statistical significance [− 0.18 $\mu$m/y (P = 0.02)] in very advanced cases of glaucoma, with an MD = − 28 dB. The differences in the mean rate of GC-IPL change between our study and these two studies may be attributable to differences in SD-OCT devices (Spectralis SD-OCT vs. OptoVue SD-OCT). In addition, Bowd et al.^5^ obtained images only twice in approximately two years and Belghith et al.^32^ performed SD-OCT scanning annually over 3.5 years. However, only the patients who underwent SD-OCT scanning at least four times during the follow-up period participated in this study. These differences in analysis strategies may contribute to the different study results.

Recent longitudinal studies reported that VD measurements are useful for evaluating glaucomatous progression, particularly in cases of severe disease^19,20^. Shin et al.^19^ revealed that monitoring the rate of longitudinal cpVD loss may help detect glaucomatous progression in moderate-to-advanced glaucomatous eyes. They examined the rate of longitudinal cpVD and cpRNFLT and their association with VF progression in 158 POAG eyes with different levels of glaucoma severity. Progressors had a faster rate of change in cpVD than non-progressors, regardless of glaucoma stage, whereas the rate of cpRNFLT thinning in progressors did not differ with non-progressors at a moderate-to-advanced stage of glaucoma. Hou et al.^20^ compared the change rate of GCCT and mVD which was measured within 3 × 3-mm^2^ scan area in healthy, pre-perimetric glaucoma and POAG eyes. They reported that POAG eyes in the advanced stage showed faster decreases in mVD than GCCT and mVD loss were significantly correlated with glaucoma severity, in contrast to the GCCT thinning.

In our event-based analysis, we used the practical tolerance limits derived from healthy eyes for defining VD and thickness parameters progression. The tolerance limits were $\pm$ 6.50 $\mathrm{\mu m}$ for cpRNFLT, $\pm$ 4.12 $\mathrm{\mu m}$ for GCCT, $\pm$ 5.16 % for cpVD, and $\pm$ 4.96 % for VD. These measurements showed excellent long-term test–retest reproducibility with ICCs ranging from 0.926–0.991 in all four parameters (Table 3). The agreement between progression detection by OCTA-derived parameters-VF progression was stronger (“substantial”) than that of OCT-derived parameters-VF progression (“slight”) (Table 4). These findings are in line with the results of our trend-based analysis in which VD parameters had a faster rate of change than structural parameters in advanced stages of glaucoma. Previous studies have reported that various SD-OCT devices showed good repeatability and reproducibility. Benjamin et al.^33^ evaluated the test–retest variability of RNFL thickness measurements using Spectralis SD-OCT in 50 normal eyes on a single day and found 0.97 for ICC, 1.7 % for COV, and 4.95 $\mathrm{\mu m}$ for the test–retest variability. Garas et al.^34^ evaluated reproducibility of cpRNFLT using RTVue-100 in 14 normal eyes and reported 0.99 for ICC, 2.6 % for COV and 4.3 $\mathrm{\mu m}$ for the test–retest variability. Kim et al.^35^ investigated the long-term reproducibility of RNFL and GCIPL thickness with Cirrus SD-OCT in clinically stable POAG patients and reported 0.969 (ICC), 3.1 % (COV), and 6.56 $\mathrm{\mu m}$ (test–retest variability) for RNFL thickness and 0.989 (ICC), 2.0 % (COV), and 4.02 $\mathrm{\mu m}$ (test–retest variability) for GC-IPL thickness. Recent studies have reported repeatability and reproducibility of OCTA-derived VD measurements. Jayasree et al.^16^ reported repeatability of peripapillary and macular VD in 30 normal eyes using OCTA performed the same day. The mean ICC of the peripapillary vessel density was 0.86, and the ICC of whole enface macular vessel density was 0.87. The test–retest variability of OCT and OCTA-measured parameters varied in studies depending on the study design, interval time, and study population. However, our study results showed ICC and COV levels similar with previous studies within an acceptable range.

We found that the number of eyes with VF progression (17 eyes, 22.7%) was higher than that of structural progression (five eyes, 6.7 % for cpRNFL thickness and six eyes, 8.0 % for GCC thickness) in an event-based analysis. (Fig. 1) This is comparable to previous studies in which functional loss was faster than the structural loss at late-stage glaucoma^36,37^. In addition, the number of eyes with VD progression (11 eyes, 14.7 % for cpVD and 10 eyes, 13.3 % for mVD) was higher than that of the thickness parameters (Fig. 1). This result implies that VD progression may be more apparent than structural progression in advanced glaucoma.

The temporal relationship between VD reduction and VF progression is noteworthy. Among the 17 patients with VF progression, eight patients showed VD reduction during the follow-up. The coincidence of mVD reduction and VF progression (five eyes, 62.5 %) was greater than that of cpVD reduction and VF progression (two eyes, 25.0 %). This implies that mVD change may have higher temporal concordance with VF progression than cpVD. However, the number of cases was too small for a conclusion, and this finding should be investigated in future research with more subjects.

Our study has several strengths. First, the present study included relatively larger number of advanced glaucoma eyes compared to previous study of Hou et al.^20^ which included only 5 advanced OAG eyes. Moreover, our study investigated larger 6 × 6-mm^2^ macular scans, which have higher diagnostic accuracy and more information about vascular change of macular area compared to 3 × 3-mm^2^ scans^38,39^. Lastly, our study explored the association between vascular and functional glaucomatous progression via the event-based analyses. The trend-based analysis can provide the rate of change over time, but it requires a longer follow-up and a greater number of tests. Since OCT-A is a relatively newly adapted device, assessment of accurate rates can be limited. In contrast, an event-based analysis is sensitive for detecting progression with fewer tests and greater variability during the follow-up^40,41^. Moreover, the event-based analysis can reflect “real-world” longitudinal data.

There are some limitations to this study. First, we had a short follow-up period (2.3 $\pm$ 1.0 years) and a small number of OCT and OCTA tests (4.5 ± 1.0 visits). Second, the number of VF progressed eyes was small (17 eyes, 22.7 %). Analysis with greater number eyes with VF progression may have provided more robust outcomes. Further large-scale study is warranted in the future. Third, we used different devices for measurements of cpRNFLT and cpVD. The Spectralis OCT and AngioVue were used for cpRFNLT and cpVD measurements, respectively. There were differences in image areas and imaging conditions, which may make it difficult to interpret and directly compare the results. However, cpRNFLT measurement area of the Angiovue (Optovue Inc) is also different with cpVD measurement area, which 3.45-mm radius ring for cpRNFLT and 4.5 × 4.5-mm^2^ scans for cpVD. Therefore, different image area derived from different devices might not have had a great impact on our results. Fourth, healthy eyes were included in a university hospital setting, which may have different characteristics than eyes observed in the general population. The tolerance limit derived from healthy eyes should be cautiously applied to a clinically relevant population. Fifth, we did not evaluate the potential confounding effects of systemic conditions, IOP, and blood pressure-lowering medications on VD measurements. As ocular hypotensive eyedrops or systemic hypotensive medications may affect ocular blood flow, they may affect the OCTA measurements. Therefore, our results should be interpreted with a possibility of confounding effects of topical and/or systemic hypotensive medications. Lastly, as our study evaluated a single ethnic group, our data may not be generalizable to the overall population. Further large-scale studies involving various ethnicities will be needed.

In conclusion, we explored glaucomatous progression in advanced-stage glaucoma by both trend and event-based analysis. In the trend-based analysis, the circumpapillary and macular VD reduction rates in the VF progression group were significantly faster than in the stable group, whereas the cpRNFLT and GCCT reduction rate did not show a statistical difference between progressors and non-progressors. In the event-based analysis, circumpapillary and macular VD showed stronger agreement with VF than agreement between thickness parameter and VF progression. These findings suggest that OCTA-derived VD parameters have significantly greater progression detection capabilities than OCT-derived structural parameters in advanced glaucomatous eyes.

## Data Availability

The datasets generated and analyzed during the current study are available from the corresponding author on reasonable request.
